# RBM15 Mediated m6A Modification of SRSF1 Inhibits Cuproptosis in Non‐Small Cell Lung Cancer by Mediating ATP7B Alternative Splicing

**DOI:** 10.1002/kjm2.70098

**Published:** 2025-09-09

**Authors:** Shan‐shan Mao, Dong‐yu Wu, Rong‐hua Cui, Xiao‐zhen Cheng

**Affiliations:** ^1^ Ward 1 of the Radiotherapy Department The First Clinical College, The First Affiliated Hospital, Hainan Medical University Haikou Hainan People's Republic of China; ^2^ Department of Medical Oncology Haikou People's Hospital Haikou Hainan People's Republic of China

**Keywords:** alternative splicing, cuproptosis, NSCLC, RBM15, SRSF1

## Abstract

Inhibition of cuproptosis contributes to the development of non‐small cell lung cancer (NSCLC). The expression of RNA‐binding motif protein 15 (RBM15) is upregulated in NSCLC. Nonetheless, its relationship with cuproptosis remains unclear. This study aimed to explore the role of RBM15 in regulating cuproptosis in NSCLC. A549 cells were treated with elesclomol (ES‐Cu) and tetrathiomolybdate (TTM) to induce or inhibit cellular cuproptosis. EdU, CCK‐8, Transwell assays, and flow cytometry were used to detect cellular phenotypes. The expression levels of relevant genes and proteins were analyzed using RT‐qPCR and western blotting. RIP and MeRIP were utilized to investigate the interaction of RBM15 and YT521‐B homology domain family‐3 (YTHDF3) with serine/arginine splicing factor 1 (SRSF1). The effect of the RBM15/m6A/SRSF1/ATP7B axis on tumor growth was evaluated using tumor xenografts in nude mice. Copper levels were assessed using commercially available kits. In NSCLC cells, RBM15 suppression inhibited proliferation and invasion while promoting cuproptosis; however, treatment with TTM (copper chelators) reversed the effect of sh‐RBM15. ES‐Cu treatment inhibited cell proliferation and invasion, and RBM15 knockdown further promoted the effect of ES‐Cu, but upregulated RBM15 reversed the regulatory effect of ES‐Cu. Mechanistically, RBM15 promoted the m6A modification of SRSF1 by recruiting YTHDF3. Increased SRSF1 enhanced ATPase copper‐transporting beta (ATP7B) exon 21 splicing. Furthermore, SRSF1 promoted cell proliferation and invasion and inhibited cuproptosis by regulating ATP7B alternative splicing. Finally, we verified that RBM15 promoted tumor growth by mediating SRSF1 in vivo. In short, RBM15‐mediated m6A modification enhanced SRSF1 stability, and SRSF1 promoted ATP7B alternative splicing to inhibit cuproptosis, thereby promoting NSCLC cell proliferation and tumor growth.

**Abbreviations**
ATP7B: ATPase copper‐transporting beta
BIN1: bridging integrator 1
CCK‐8: Cell Counting Kit‐8
DLD: dihydrolipoamide dehydrogenase
ES‐Cu: elesclomol
FBS: fetal bovine serum
IGF2BP2: insulin‐like growth factor 2 mRNA‐binding protein 3
IHC: immunohistochemical
m6A: N6‐methyladenosine
Me‐RIP: methylated RNA immunoprecipitation
METTL3: methyltransferase 3
MYBL2B: B‐Myb
NSCLC: non‐small cell lung cancer
PBS: phosphate‐buffered saline
RBM15: RNA‐binding motif protein 15
RIP: RNA immunoprecipitation
RT‐qPCR: quantitative real‐time PCR
SLC31A1: solute carrier family 31 member 1
SR: serine/arginine
SRSF1: serine/arginine splicing factor 1
TMBIM6: transmembrane BAX inhibitor motif containing 6
WT: wild‐type
YTHDF3: YT521‐B homology domain family

## 1 Introduction

Lung cancer is a widespread and lethal malignant tumor globally [1]. It is mainly classified into small cell lung cancer and non‐small cell lung cancer (NSCLC), with NSCLC accounting for more than 85% of all lung cancer cases [2]. In recent years, immunotherapy has shown significant potential in NSCLC treatment [3]. Nevertheless, it imposes a substantial economic burden on patients, and the prognosis and survival rates require improvement. Therefore, the development of novel targeted therapies is crucial to enhance the survival rate of patients with NSCLC.

Cuproptosis is characterized by the accumulation of copper ions, which cause the abnormal aggregation of thiolated proteins within cells [4]. Previous research has revealed that a decrease in serum copper concentration is associated with an increased likelihood of lung cancer occurrence and progression [5]. Disulfiram has been shown to potentiate the cuproptosis‐mediated suppression of NSCLC cell viability [6]. Therefore, an in‐depth study of copper‐mediated cell death mechanisms could provide new breakthroughs in lung cancer treatment. N6‐methyladenosine (m6A) modification is a highly conserved RNA modification that plays a key role in NSCLC. Notably, RNA‐binding motif protein 15 (RBM15) is a crucial regulator of m6A modification and is highly expressed in NSCLC [7]. Silencing RBM15 has been demonstrated by previous studies to significantly reduce the invasion, migration, and proliferation of NSCLC cells [8]. Furthermore, extensive research has revealed a significant correlation between genes implicated in m6A methylation and those linked to cuproptosis in sepsis‐induced cardiotoxicity [9]. In addition, METTL16 promotes cuproptosis through m6A modification on FDX1 mRNA in gastric cancer [10]. Nonetheless, the relationship between RBM15 and cuproptosis has not been reported.

The serine/arginine (SR)‐rich protein family, especially serine/arginine splicing factor 1 (SRSF1), plays an indispensable role as a key regulator in splicing, and various SR proteins exhibit oncogenic properties in cancer development [11]. SRSF1 has been confirmed to be abnormally expressed in lung adenocarcinoma and squamous cell lung cancer [12]. Additionally, SRSF1 promotes NSCLC progression by reducing the expression of bridging integrator 1 (BIN1) via alternative splicing [13]. Interestingly, multiple m6A modification sites have been found in SRSF1 mRNA using SRAMP. Therefore, we speculate that RBM15 may mediate the m6A modification of SRSF1 mRNA to regulate carcinogenesis in NSCLC. ATPase copper‐transporting beta (ATP7B) is an important cell membrane protein that controls copper metabolism and homeostasis [6, 14]. High ATP7B expression enhances the platinum‐based chemotherapy response in patients with NSCLC [15]. Disulfiram also induces cuproptosis in NSCLC by reducing ATP7B levels [6]. The results of the ESEfinder analysis indicated that SRSF1 mediated the alternative splicing of ATP7B exon 21. However, whether SRSF1 regulates NSCLC progression by mediating ATP7B alternative splicing requires further elucidation.

In this study, we discovered that RBM15‐mediated m6A modification of SRSF1 inhibited cuproptosis via ATP7B alternative splicing in NSCLC; which may provide a new therapeutic target for NSCLC treatment.

## 2 Materials and Methods

### 2.1 Cells Culture and Treatment

Human non‐small lung cancer cell lines (H1299, H460, H1915, and A549) were obtained from the American Type Culture Collection (Manassas, VA, USA). The cells were cultivated in RPMI 1640 medium containing 10% fetal bovine serum (FBS; Gibco, MA, USA) and maintained at 37°C under 5% CO_2_. After 48 h, 100 μL of the cells were transferred to 96‐well plates at a density of 5 × 10^3^ cells per well. A549 cells were exposed to elesclomol (ES‐Cu; HY‐12040, MCE, California, USA) at 100, 200, 300, 400, and 500 nM (1:1 ratio), Z‐VAD‐FMK (Z‐Vad; HY‐16658B, MCE) at 30 μM, necrostatin‐1 (Nec‐1; HY‐15760, MCE) at 10 μM, ferrostatin‐1 (Fer‐1; HY‐100579, MCE) at 10 μM, and tetrathiomolybdate (TTM; 323446, Sigma–Aldrich, MO, USA) at 20 μM for 2 h.

### 2.2 Lentiviral Vector Construction and Transfection

Lentivirus vectors for RBM15 and SRSF1 knockdown (sh‐RBM15 and sh‐SRSF1) were obtained from GenePharma (Shanghai, China). Lipofectamine 3000 (Invitrogen, CA, USA) was utilized to transfect 293 T cells with a combination of 10 μg of pPACK Packaging Plasmid Mix, 2 μg expression vector, and shRNA (Cellecta, CA, USA) incubated for 48 h. The supernatant was harvested and centrifuged at 4°C for 10 min, concentration and purification. A549 cells were transfected with the lentivirus for 72 h and screened for stable transformation using puromycin (4 μg/mL). The cells were collected for functional assays.

### 2.3 Cell Counting Kit‐8 Assay

Cell viability was determined using a Cell Counting Kit‐8 (CCK‐8) kit (Yeasen, Shanghai, China). In brief, 5 × 10^3^ A549 cells were seeded into each well of 96‐well plates and incubated at 37°C for 12, 24, 36, and 48 h. Following this, 100 μL of CCK‐8 reagent was introduced to each well, and the plates were further incubated for 1 h. Subsequently, a microplate reader (Bio‐Rad, CA, USA) was used to measure optical density at a wavelength of 450 nm.

### 2.4 EdU Assay

A549 cells were plated into a 96‐well plate at a density of 2 × 10^5^ cells per well and incubated for 24 h. Following the protocol of the EdU kit (C0071s; Beyotime, Shanghai, China), 100 μL of EdU medium was added to each well for 2 h and then fixed with 100 μL of 4% paraformaldehyde for 30 min. Subsequently, the cells in each well were stained with 100 μL of fluorescent dye and reaction solution for 30 min. Imaging was performed using a fluorescence microscope.

### 2.5 Copper Microplate Assay

Intracellular copper ions were detected using a copper microplate assay kit (Absin Bioscience, Shanghai, China). A549 cells were collected and lysed. Following centrifugation, the supernatant was removed and 1 mL of assay buffer was added. The cells were sonicated at 20% power for 3 s, followed by a 10 s interval, and the sonication process was repeated three times. Subsequently, the mixture was centrifuged at 4°C for 10 min, and the resulting supernatant was collected and analyzed using a microplate reader at 605 nm.

### 2.6 Transwell Assay

A volume of 100 μL containing 2 × 10^5^ cells per well was introduced into the upper chamber of a Transwell plate that had been precoated with Matrigel (BD Biosciences, CA, USA). 200 μL serum‐free medium was added to the chamber. Meanwhile, the lower chamber was filled with 400 μL of complete medium supplemented with 10% FBS. After 24 h of incubation, the cells were fixed with paraformaldehyde, stained with 0.4% crystal violet, photographed, and carefully observed under a microscope.

### 2.7 Flow Cytometry

The collected cells were washed three times with cold PBS. Next, 5 μL of PI and 5 μL Annexin V‐FITC were added into 100 μL of the cell (2 × 10^5^) solution; then, it was incubated at 25°C for 15 min to avoid the light. The flow cytometry (BD Biosciences) was employed to analyze the cell apoptosis.

### 2.8 Western Blotting

Total protein was extracted from cells and tissues, and quantified using the BCA protein assay kit (Epizyme Biotech, Shanghai, China). Proteins were separated by sodium dodecyl sulfate‐polyacrylamide gel electrophoresis and transferred to a PVDF membrane, which was blocked using a solution containing BSA and 5% skim milk. Subsequently, the membranes were incubated overnight at 4°C with primary antibodies against YT521‐B homology domain family‐3 (YTHDF3) (1:1000, Abcam, ab220161), ATP7B (1:1000, ab124973), DLD (1:2000, Proteintech, 16431‐1‐AP), SRSF1 (1:1000, Invitrogen, PA5‐30220) and GAPDH (1:1000, Abcam, ab9485). The membrane was then incubated for 1 h with a goat anti‐rabbit IgG secondary antibody (1:1000, Abcam, ab205718). Protein bands were detected using an enhanced chemiluminescence kit (#34580; Thermo Fisher Scientific, MA, USA) on a ChemiDoc XRS System (Bio‐Rad).

### 2.9 Quantitative Real‐Time PCR (RT‐qPCR)

RNA was extracted from the cells using TRIzol reagent (Invitrogen). Subsequently, 1 μg RNA was reversed and transcribed into cDNA by utilizing PrimeScript RT reagent Kit (Takara, Beijing, China). The expression levels of RBM15, YTHDF3, ATP7B, and SRSF1 were assessed using the SYBR qPCR Master Mix (Vazyme, Jiangsu, China), with GAPDH as the internal control. The relative RNA expression was calculated using 2^−ΔΔCt^. The primer sequences were shown in Table 1.

**TABLE 1:** Primer sequences used in this study.

Gene name	Forward primer (5′‐3′)	Reverse primer (5′‐3′)
YTHDF3	AGCCAGACAAATCAGAGTAACAG	CCTAATGCCCCAGGTTGACT
ATP7B	GTCACACCCTGCTTGGATTT	CCTGATGGGCCTATTTCTCA
ATP7B‐s	CCACACCATGGGACCAGGTC	GTGGTGAGTGGAGGCAAGTC
ATP7B‐l	TGGTGTTGTTCAGAGATACT	CCTCCCTTGATGAGGATGCC
RBM15	TCGCTTTGGAGTCATCACAG	GGGTGGTGGGTGTAGCTTTA
SRSF1	CCTTCGTTGAGTTCGAGGAC	ATCCTGCCAACTTCCACTTG
GAPDH	CTGACTTCAACAGCGACACC	GTGGTCCAGGGGTCTTACTC

### 2.10 RNA Binding Protein Immunoprecipitation Assay (RIP)

RIP was performed following the instructions of the RNA immunoprecipitation kit (GENESEED, Guangzhou, China). In brief, cells were lysed and incubated with anti‐YTHDF1 (1:30, Abcam, ab220162), YTHDF2 (1:30, Abcam, ab220163), YTHDF3 (1:30, Abcam, ab220161), YTHDC1 (1:50, Abcam, ab264375), YTHDC2 (1:30, Abcam, ab220160) and SRSF1 (1:1000, Invitrogen, PA5‐30220) or anti‐IgG (1:100, ab205718, Abcam) for 4 h at 4°C. The RNA‐protein complexes were captured using protein A/G magnetic beads (Abcam, ab286842). After 1 h, the beads were washed, proteinase K was added, and immunoprecipitated RNA was isolated. RNA was extracted and purified, and RT‐qPCR analyzed the level of mRNA.

### 2.11 Methylated RNA Immunoprecipitation Sequencing (MeRIP‐Seq)

RNA eluted from the m6A RNA immunoprecipitation experiments was processed with RNasin (AM2694; Invitrogen) according to the manufacturer's instructions. The library was prepared using the Illumina TruSeq Stranded mRNA Library Prep Kit, with immunoprecipitated RNA samples and input RNA controls processed separately, according to a published protocol. Sequencing was performed on an Illumina HiSeq platform under paired‐end 2 × 100‐cycle sequencing mode.

### 2.12 Methylated RNA Immunoprecipitation (MeRIP)

Dynabeads mRNA (#61006; Invitrogen) were used to extract and purify mRNA. Then the anti‐m6A (Abcam, ab208577) was coupled to protein A/G magnetic beads for 4 h. After washing, DNA‐free RNA was treated with RNase and protease inhibitors and incubated for 2 h. The interacting RNA was isolated using an RNA Clean & Concentrator kit (ZYMO Research, CA, USA, R1016) and detected by RT‐qPCR. We used a normalized input (% input) approach to determine relative SRSF1 m6A levels: relative enrichment = 2^Ct[IP]‐Ct[input]^, where Ct[IP] is the cycling threshold for immunoprecipitation samples and Ct[input] is the cycling threshold for input samples.

### 2.13 Detection of mRNA Stability

5 μg/mL Actinomycin D (GlpBio Technology, CA, USA, #GC16866) treated cells for 0, 2, 4, and 6 h to detect mRNA decay. RNA was subsequently extracted, and changes in SRSF1 mRNA levels were detected using RT‐qPCR.

### 2.14 Gel Detection of mRNA Levels

To detect the alternative splicing pattern of ATP7B mRNA, the extracted total RNA was reverse‐transcribed, and 1 μL of the cDNA was used as the template for PCR amplification. The products were subjected to agarose gel electrophoresis.

### 2.15 In Vivo Xenograft Mouse Model

BALB/c mice (nude, 4–5 weeks, 17–23 g, specific pathogen‐free (SPF) grade) were obtained from Hunan SJA Laboratory Animals, maintained, and fed under a 12 h light and dark cycle. Thirty‐six mice were divided into six groups for the xenograft models. Approximately 2 × 10^6^ A549 cells stably expressing targeting gene were injected subcutaneously into the axillae of the mice. Then, the mice were injected with ES‐Cu (20 mg/kg). Weekly measurements of tumor width (*W*) and length (*L*) were conducted using calipers, and tumor volume was computed using the following formula: *V* = (*W* ^2^ × *L*)/2. After injecting the mice for 5 weeks, the subcutaneous tumor was harvested and weighed. All animal experiments were approved by the Animal Care Committee and conducted according to the ethical standards of the animal experimental system approved by the Ethics Committee of Hainan Medical University (XSTS2025073).

### 2.16 Immunohistochemistry (IHC) Staining

Tissue was fixed in paraffin, then sectioned into 4 μm slices. These sections were dewaxed with standard xylene, followed by dehydration using a series of gradient alcohols. The cells were then incubated in 3% H_2_O_2_ for 30 min and rinsed with PBS. Next, the slices were blocked with normal sheep serum at 37°C for 10 min and incubated overnight with anti‐Ki‐67 (1:200, Abcam, ab15580). Next, they were washed and incubated with goat anti‐rabbit anti‐IgG antibody (1:500, Abcam, ab6112) for 1 h. Finally, DAB staining was used to capture images under a microscope.

### 2.17 TUNEL Assay

This method was based on the instructions of the TUNEL Apoptosis Detection Kit (40306ES60; Yeasen). Slices were processed as previously described, exposed to TUNEL reaction solution for 1 h at 37°C away from light, and then mounted with DAPI at 37°C for 10 min. Finally, the slices were washed several times with PBS and examined under a fluorescence microscope.

### 2.18 Statistical Analysis

Data analysis was performed using GraphPad Prism 8.0. Statistical comparisons between two groups were performed using Student's *t*‐test, and additional groups were subjected to one‐way analysis of variance (ANOVA). All experiments were replicated more than three times, and the results are reported as the mean ± standard deviation (mean ± SD). Statistical significance was set at *p* < 0.05.

## 3 Results

### 3.1 RBM15 Promoted NSCLC Cell Proliferation, Invasion, and Inhibited Cuproptosis

**FIGURE 1:**
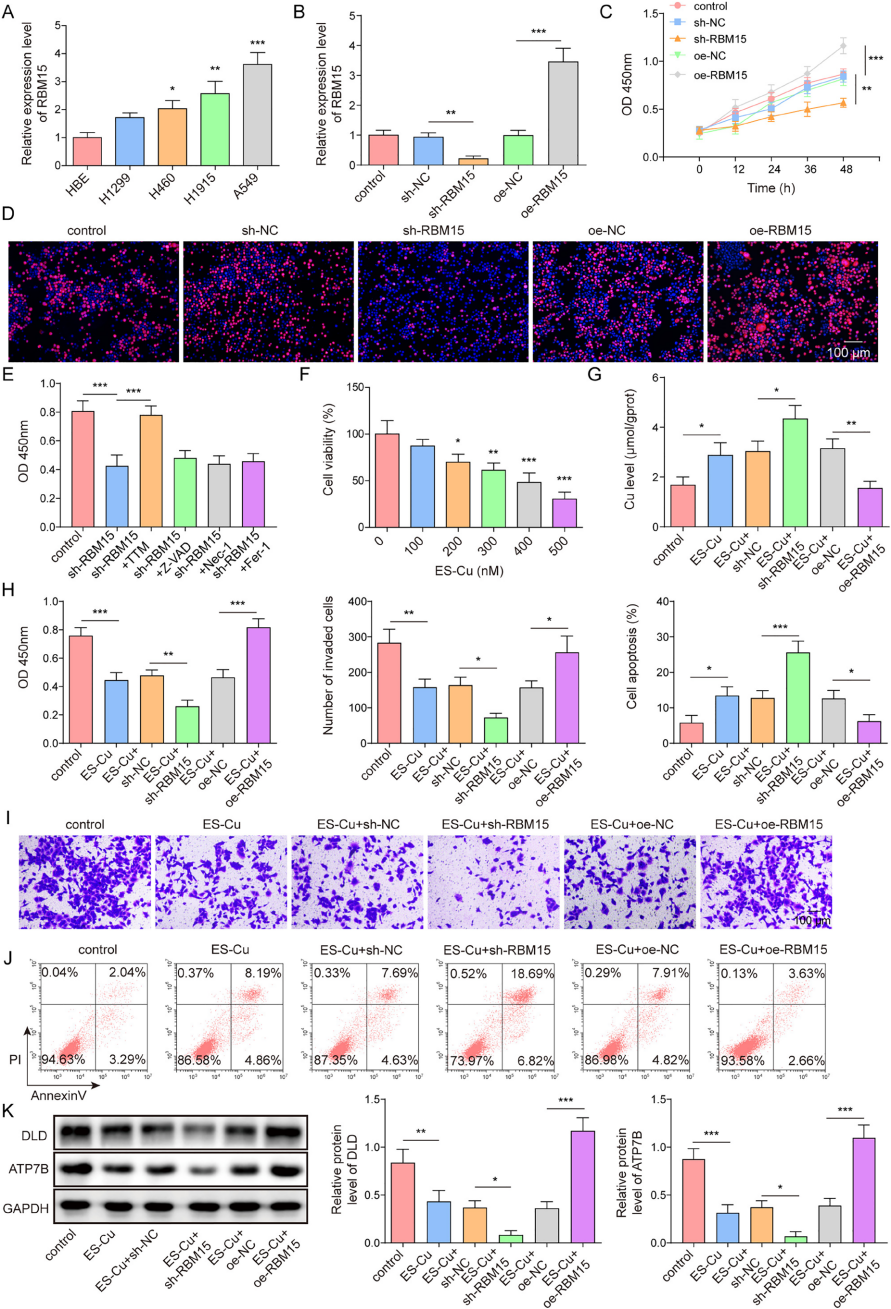
RBM15 knockdown inhibited NSCLC cell proliferation, invasion, and induced cuproptosis. (A) The expression level of RBM15 was detected through RT‐qPCR in different types of human NSCLC cells and normal cell lines. (B–D) A549 cells transfected with sh‐RBM15 or oe‐RBM15. (B) The efficiency of RBM15 knockdown or overexpression was tested by RT‐qPCR. (C) Cell viability was detected using CCK‐8 assay. (D) Proliferation ability was evaluated using EdU assay. (E) CCK‐8 assay detected the viability of A549 cells pretreated with Z‐VAD‐FMK (Z‐VAD), ferrostatin‐1 (Fer‐1), necrostatin‐1 (Nec‐1), and TTM. (F) Cells were treated with different concentrations of ES‐Cu, and cell viability was detected using CCK‐8 assay. (G–K) sh‐RBM15 was transfected into A549 cells and treated with 300 nM ES‐Cu, setting up the groups: Control, ES‐Cu, ES‐Cu + sh‐NC, ES‐Cu + sh‐RBM15, ES‐Cu + oe‐NC, ES‐Cu + oe‐RBM15. (G) Copper microplate assay assessed copper levels. (H) Cell viability was detected using CCK‐8 assay. (I) Transwell assay was used to evaluate cell invasion. (J) Levels of apoptosis were evaluated by flow cytometry. (K) DLD and ATP7B protein expression were detected by western blotting. Data are presented as mean ± SD (*n* = 3, *p < 0.05, **p < 0.01, ***p < 0.001).

To clarify the role of RBM15 in lung cancer, we measured RBM15 expression in various lung cancer cell lines. The results indicated that RBM15 was highly expressed, particularly in A549 cells (Figure 1A). Therefore, A549 cells were selected for the subsequent experiments. We constructed and validated a cellular model of RBM15 overexpression or knockdown; the results showed that RBM15 expression was decreased or increased after sh‐RBM15 or oe‐RBM15 treatment (Figure 1B). Furthermore, RBM15 knockdown significantly reduced cell viability and proliferation compared to those in the control group, while overexpression of RBM15 promoted cell viability and proliferation (Figure 1C,D). Subsequently, A549 cells were treated with a range of cell death inhibitors, including the apoptosis inhibitor Z‐VAD, ferroptosis inhibitor Fer‐1, necrotic cell death inhibitor Nec‐1, and cuproptosis inhibitor TTM. The results showed that only TTM significantly restored the reduction in cell viability caused by RBM15 knockdown, whereas the other inhibitors had no significant effect (Figure 1E), strongly suggesting RBM15 might be involved in the development of lung cancer by regulating cuproptosis. Then, A549 cells were exposed to various concentrations of ES‐Cu. ES‐Cu is a potent copper‐ion carrier that inhibits cell viability, promotes apoptosis, and induces cuproptosis [16]. As the concentration of ES‐Cu increased, cell viability gradually decreased to approximately 50% at 300 nM (Figure 1F). Therefore, 300 nM ES‐Cu was selected as the optimal dose for subsequent experiments. We then measured the changes in intracellular copper levels and found that ES‐Cu treatment significantly elevated these levels compared to those in the control group, and RBM15 knockdown further increased intracellular copper levels, but the intracellular copper content was decreased by RBM15 overexpression (Figure 1G). CCK‐8 and Transwell assay data indicated that RBM15 knockdown enhanced ES‐Cu inhibition of cell proliferation and invasion. In contrast, RBM15 overexpression attenuated the inhibitory effect of ES‐Cu (Figure 1H,I). RBM15 knockdown increased apoptosis induced by ES‐Cu, while RBM15 overexpression inhibited ES‐Cu‐induced apoptosis (Figure 1J). Finally, as shown in Figure 1K, ES‐Cu treatment inhibited the expression of dihydrolipoamide dehydrogenase (DLD) and ATP7B, whereas RBM15 knockdown further reduced the expression levels of DLD and ATP7B. However, overexpression of RBM15 rescued the expression of DLD and ATP7B induced by ES‐Cu. In addition, we used TTM to treat A549 cells. The results showed that TTM treatment effectively reversed the decrease in cell viability induced by RBM15 knockdown (Figure S1A,B). RBM15 knockdown promoted apoptosis, which was partially reversed by TTM (Figure S1C). Notably, TTM treatment effectively reversed the suppression of DLD and ATP7B expression induced by RBM15 silencing (Figure S1D). In summary, RBM15 knockdown worked synergistically with ES‐Cu treatment to inhibit the proliferation and invasion of A549 cells.

### 3.2 RBM15 Stabilized the Expression of SRSF1 mRNA by YTHDF3‐m6A

**FIGURE 2:**
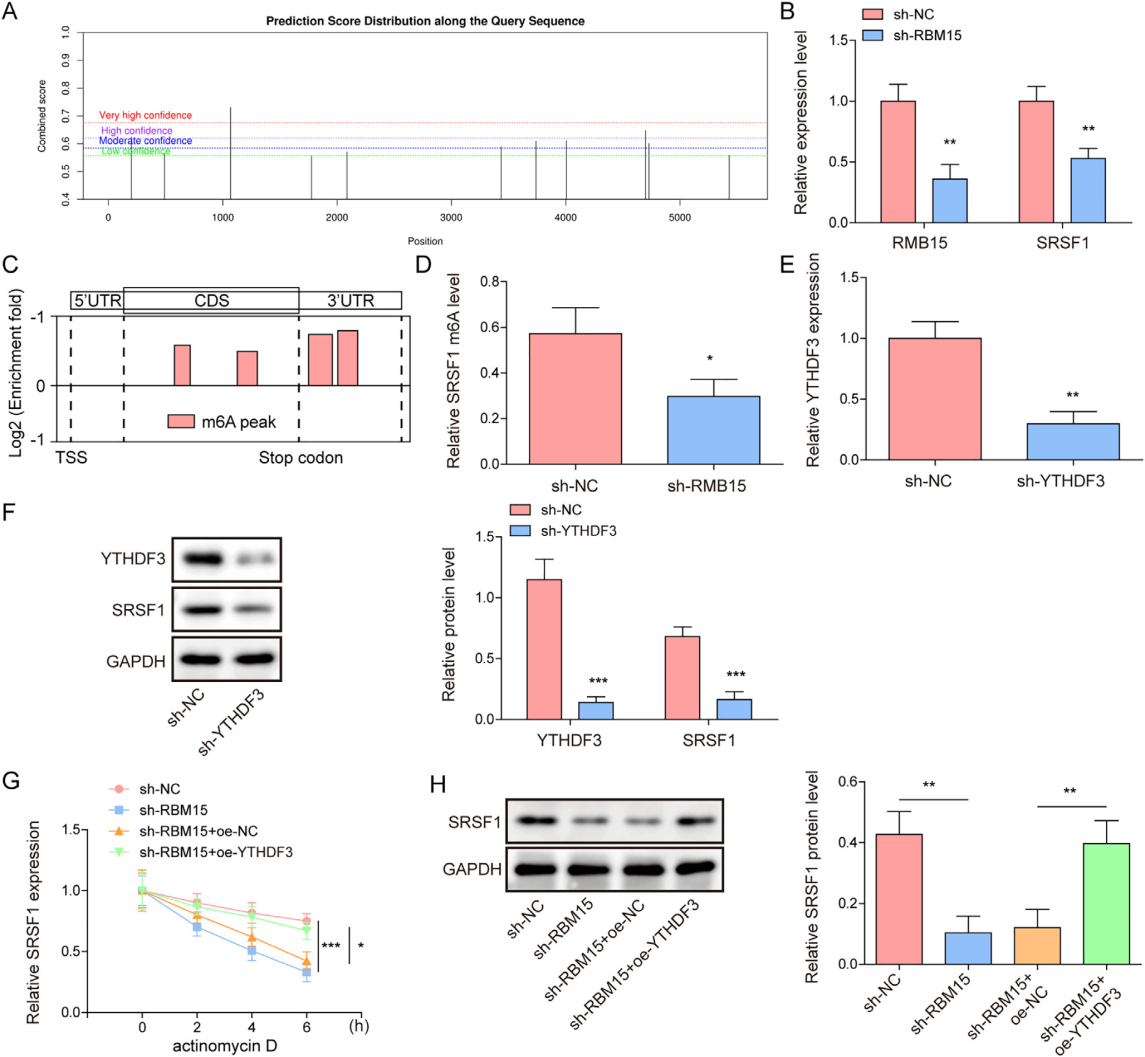
RBM15 stabilized the expression of SRSF1 mRNA by YTHDF3‐m6A. (A) Prediction of potential m6A binding sites within the SRSF1 mRNA. (B) RBM15 and SRSF1 mRNA levels after RBM15 knockdown were detected by RT‐qPCR. (C‐D) m6A modification of SRSF1 mRNA was measured using MeRIP. (E) The efficiency of YTHDF3 knockdown in A549 cells was evaluated through RT‐qPCR. (F) YTHDF3 and SRSF1 protein levels in YTHDF3 knockdown cell lines were measured using western blotting. (G) RT‐qPCR was conducted to assess the stability of SRSF1 after RBM15 knockdown or YTHDF3 overexpression. (H) Western blotting was used to detect SRSF1 protein expression. Data are presented as mean ± SD (*n* = 3, *p < 0.05, **p < 0.01, ***p < 0.001).

We predicted the m6A modification of SRSF1 using the SRAMP method and identified potential m6A modification sites on the SRSF1 mRNA (Figure 2A). We observed that RBM15 knockdown inhibited the expression of RBM15 and SRSF1 and significantly decreased the m6A level of SRSF1 mRNA (Figure 2B–D). In addition, RIP experiments were performed to verify the binding between the m6A recognition proteins (YTHDF1/2/3, YTHDC1/2) and SRSF1 mRNA. As shown in Figure S2, the results displayed that the inhibition of YTHDF3 binding to SRSF1 was more significant in the case of RBM15 knockdown. Therefore, we selected YTHDF3 as the m6A recognition protein of SRSF1. Next, we knocked down YTHDF3 in A549 cells and assessed the knockdown efficiency (Figure 2E). YTHDF3 knockdown resulted in the downregulation of the protein expression levels of SRSF1 and YTHDF3 (Figure 2F). As shown in Figure 2G, RBM15 depletion reduced the half‐life of SRSF1, whereas YTHDF3 overexpression increased its half‐life of SRSF1. Western blotting showed that RBM15 knockdown decreased the SRSF1 protein expression, which was partially restored by YTHDF3 overexpression (Figure 2H). These results collectively reveal new mechanisms by which RBM15 via YTHDF3 regulates the stability of SRSF1 mRNA.

### 3.3 RBM15 Mediated the m6A Modification of SRSF1 to Promote Cell Proliferation and Invasion and Inhibit Cuproptosis

**FIGURE 3:**
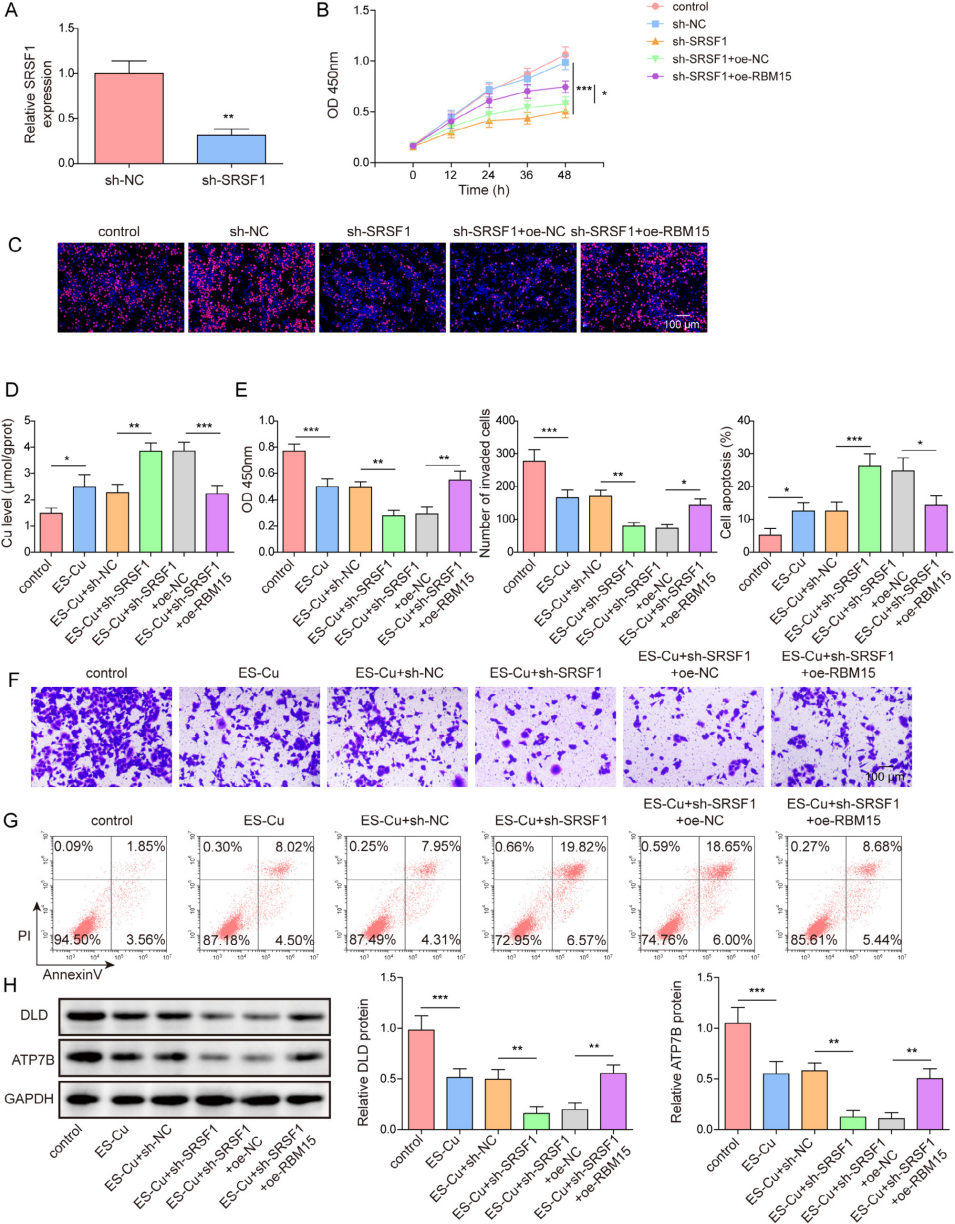
RBM15 mediated the m6A modification of SRSF1 to promote cell proliferation and invasion and inhibit cuproptosis. Sh‐SRSF1 and/or oe‐RBM15 were transfected into A549 cells. (A) The efficiency of SRSF1 knockdown was tested by RT‐qPCR. (B) Cell viability was detected using CCK‐8 assay. (C) Proliferation ability was evaluated using EdU assay. (D–H) sh‐SRSF1 and/or oe‐RBM15 were transfected into A549 cells, and A549 cells were then treated with 300 nM ES‐Cu, setting up the groups: Control, ES‐Cu, ES‐Cu + sh‐NC, ES‐Cu + sh‐SRSF1, ES‐Cu + sh‐SRSF1 + oe‐NC, ES‐Cu + sh‐SRSF1 + oe‐RBM15. (D) Copper levels were assessed using copper microplate assay. (E) Cell viability was detected using CCK‐8 assay. (F) Cell invasion ability was evaluated using Transwell assay. (G) Levels of apoptosis were evaluated by flow cytometry. (H) Western blotting was used to detect DLD and ATP7B protein expression. Data are presented as mean ± SD (*n* = 3, *p < 0.05, **p < 0.01, ***p < 0.001).

To further explore the role of SRSF1 and RBM15 in cellular processes, we first knocked down SRSF1. SRSF1 knockdown efficiency was confirmed by RT‐qPCR (Figure 3A). SRSF1 knockdown inhibited cell viability and proliferation, whereas RBM15 overexpression reversed these effects (Figure 3B,C). SRSF1 knockdown further promoted the ES‐Cu‐induced increase in copper levels, whereas RBM15 overexpression reversed this effect (Figure 3D). SRSF1 knockdown augmented the inhibitory effect of ES‐Cu on cell proliferation and invasion, whereas RBM15 overexpression attenuated this inhibitory effect (Figure 3E,F). Furthermore, RBM15 overexpression reversed the enhanced ES‐Cu‐induced apoptotic response caused by SRSF1 knockdown (Figure 3G). In addition, SRSF1 knockdown significantly reduced DLD and ATP7B expression, whereas RBM15 overexpression reduced this inhibitory effect (Figure 3H). These results suggested that RBM15 inhibited coproptosis and increased cell migration and invasion via SRSF1.

### 3.4 SRSF1 Mediated Variable Alternative Splicing Regulation of ATP7B Exon 21 Expression

**FIGURE 4:**
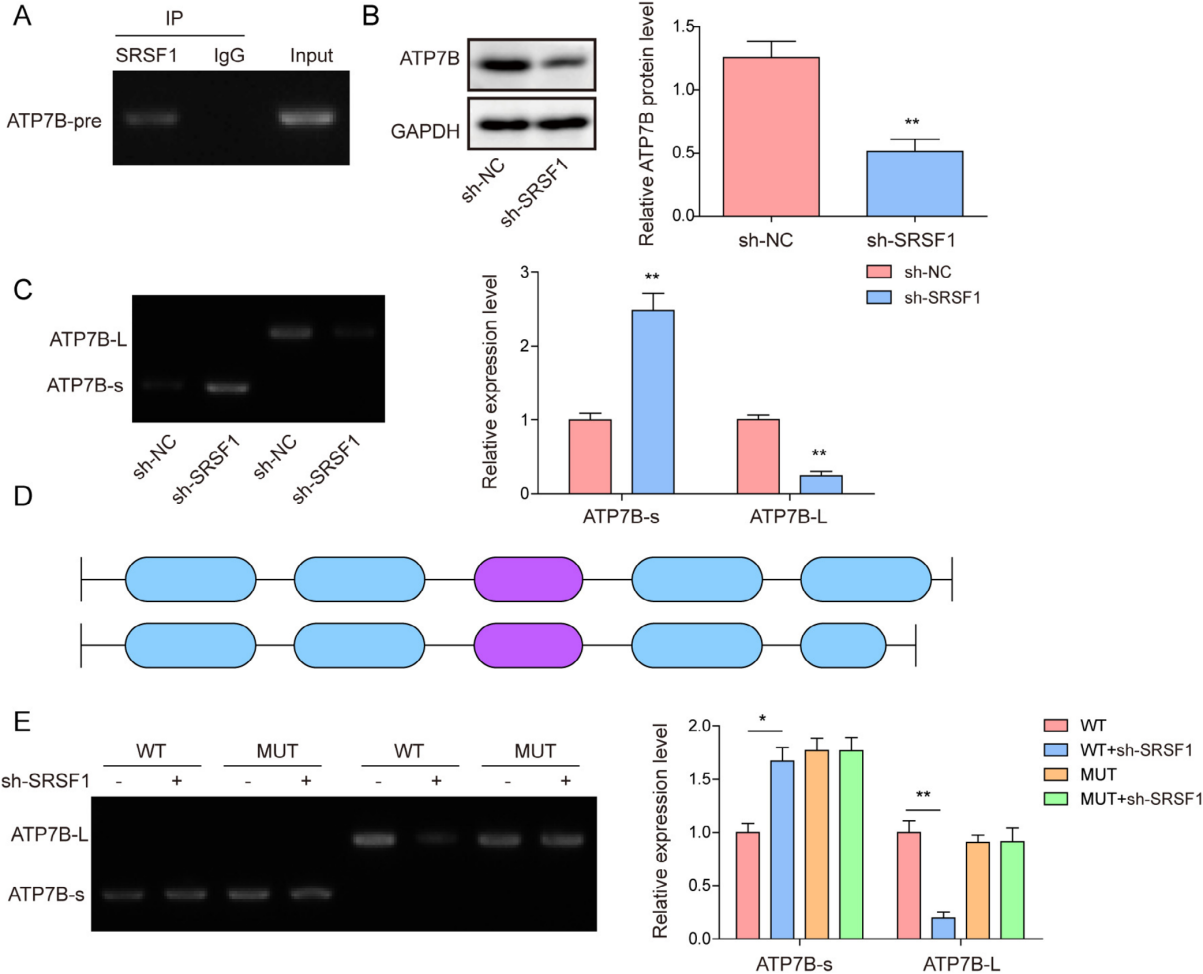
SRSF1 mediated alternative splicing regulation of ATP7B exon 21 expression. (A) RIP assay showed that the ATP7B mRNA was dropped down by SRSF1. (B) Western blot analysis was employed to detect the expression of ATP7B. (C) The change of two ATP7B transcripts following SRSF1 knockdown by agarose gel electrophoresis. (D) Schematic diagram of ATP7B alternative splicing. (E) Agarose gel electrophoresis was used to detect the change of the two ATP7B transcripts after SRSF1 knockdown. Data are presented as mean ± SD (*n* = 3, *p < 0.05, **p < 0.01).

As ATP7B can regulate copper transport and metabolism, we explored the mechanism of action of SRSF1 in NSCLC and investigated the influence of SRSF1 on ATP7B alternative splicing. These results indicated that SRSF1 could bind to ATP7B mRNA (Figure 4A). Moreover, ATP7B protein expression was significantly reduced following SRSF1 knockdown (Figure 4B). We investigated the effects of SRSF1 on the expression of these two transcripts. The results indicated that ATP7B‐L expression decreased after SRSF1 knockdown, whereas ATP7B‐S expression increased (Figure 4C). ATP7B contains 21 exons, of which exon 21 is selectively spliced to produce two transcripts (Figure 4D). Consistent with previous results, SRSF1 knockdown reduced the transcript levels of ATP7B‐L and elevated those of ATP7B‐S (Figure 4E). This suggested that SRSF1 played an essential role in regulating ATP7B alternative splicing.

### 3.5 SRSF1 Promoted Cell Proliferation, Invasion and Inhibited Cuproptosis by Regulating ATP7B

**FIGURE 5:**
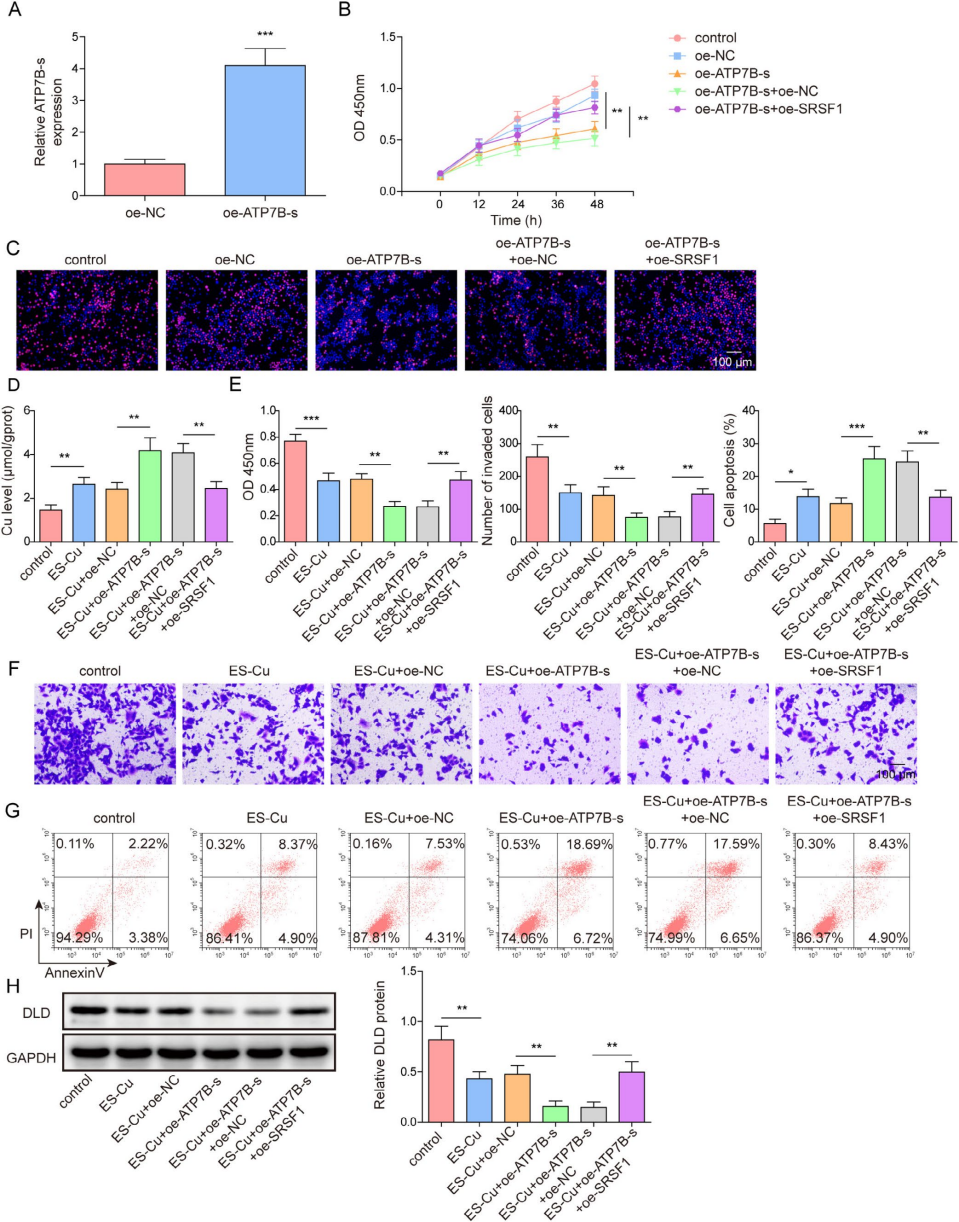
SRSF1 promoted cell proliferation, invasion, and inhibited cuproptosis by regulating ATP7B. (A–C) oe‐SRSF1 and/or oe‐ATP7B‐s were transfected into A549 cells. (A) RT‐qPCR was used to test the efficiency of ATP7B‐s overexpression. (B) Cell viability was detected using CCK‐8 assay. (C) EdU detected cell proliferation. (D–H) oe‐SRSF1 and/or oe‐ATP7B‐s were transfected into A549 cells and then treated with ES‐Cu (300 nM), setting up groups: Control, ES‐Cu, ES‐Cu + oe‐NC, ES‐Cu + oe‐ATP7B‐s, ES‐Cu + oe‐ATP7B‐s + oe‐NC, ES‐Cu + oe‐SRSF1 + oe‐ATP7B‐s. (D) Copper levels were assessed using copper microplate assay. (E) CCK‐8 assay detected the cell viability. (F) Transwell assay was utilized to evaluate the invasion ability. (G) Flow cytometry was used to evaluate cell apoptosis. (H) Western blotting was used to detect the DLD expression. Data are presented as mean ± SD (*n* = 3, *p < 0.05, **p < 0.01, ***p < 0.001).

We investigated the roles of ATP7B‐s, a short‐splice variant of ATP7B, and SRSF1 in NSCLC. First, we examined the expression levels of ATP7B‐L and ATP7B‐s in HBE1 normal lung epithelial cells and A549 cells, and found that ATP7B‐L expression was significantly upregulated in A549 cells, whereas ATP7B‐s expression was reduced (Figure S3). We overexpressed ATP7B‐s in A549 cells, and the efficiency of ATP7B‐s overexpression was detected (Figure 5A). SRSF1 overexpression reversed the inhibitory effects of ATP7B‐s overexpression on cell viability and proliferation (Figure 5B,C). ATP7B‐s overexpression further increased copper levels in ES‐Cu‐treated cells. However, simultaneous overexpression of SRSF1 reversed the effect of ATP7B‐s upregulation on copper levels (Figure 5D). ATP7B‐s overexpression increased the inhibition of ES‐Cu treatment on cell invasion, proliferation, and promotion of apoptosis, whereas SRSF1 overexpression reversed these effects of ATP7B‐s (Figure 5E–G). In addition, the results showed that ATP7B‐s decreased DLD expression in ES‐Cu‐treated cells, whereas SRSF1 overexpression increased DLD expression (Figure 5H). Furthermore, the upregulation of ATP7B‐s resulted in a decrease in cell viability and invasion, and induced apoptosis. TTM treatment reversed the effects of ATP7B‐s overexpression (Figure S4A–C). TTM weakened the inhibitory effect of ATP7B on the DLD protein levels (Figure S4D). These results suggested that SRSF1 promoted cell proliferation and invasion and inhibited cuproptosis by mediating ATP7B alternative splicing.

### 3.6 RBM15 Promoted Tumor Growth by Mediating SRSF1 In Vivo

**FIGURE 6:**
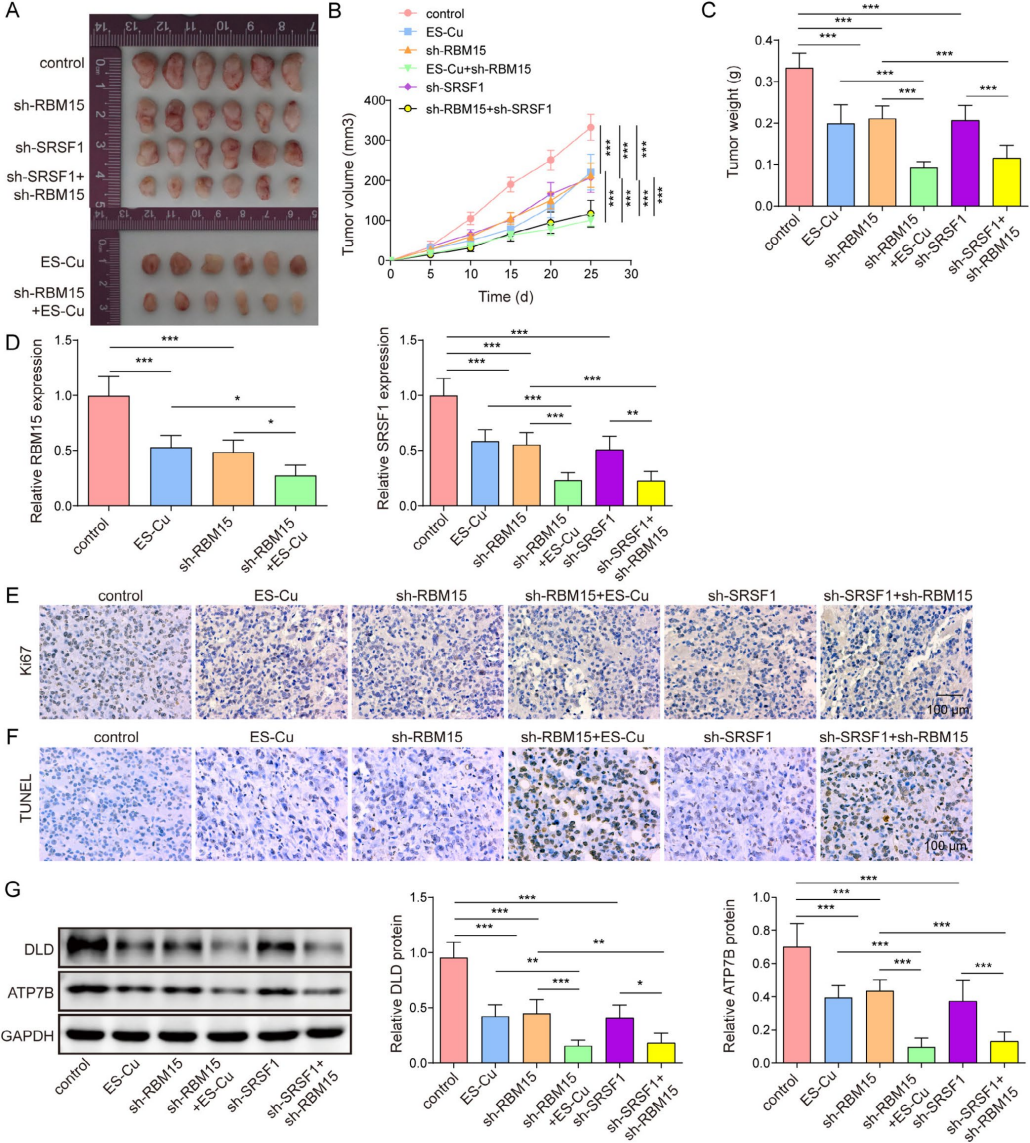
RBM15 promoted tumor growth by mediating SRSF1 in vivo. A549 cells were treated with RBM15 and/or SRSF1 and injected subcutaneously into BALB/c mice; tumor‐bearing mice were treated with ES‐Cu, setting up groups (*n* = 6/group): Control, ES‐Cu, sh‐RBM15, ES‐Cu + sh‐RBM15, sh‐SRSF1, sh‐RBM15 + sh‐SRSF1. (A) Tumor representative diagram. (B) Tumor growth curves. (C) Tumor weight of mice. (D) RBM15 and SRSF1 expression were detected by RT‐qPCR. (E) IHC detected the expression level of KI‐67. (F) Cell apoptosis was detected by TUNEL assay. (G) Western blotting was used to detect the DLD and ATP7B expression. Data are presented as mean ± SD (*n* = 6, *p < 0.05, **p < 0.01, ***p < 0.001).

We confirmed the role of RBM15‐mediated SRSF1 m6A modification in vivo. A549 cells transfected with sh‐NC, sh‐RBM15, or sh‐SRSF1 were subcutaneously injected into nude mice. Tumor‐bearing mice were treated with ES‐Cu. The weight and size of the tumors in the sh‐RBM15 and sh‐SRSF1 groups were smaller than those in the control group. ES‐Cu treatment inhibited tumor growth, whereas sh‐RBM15 enhanced its inhibitory effect. Furthermore, combined RBM15 and SRSF1 knockdown further inhibited tumor growth (Figure 6A–C). We found that RBM15 knockdown or ES‐Cu treatment significantly reduced RBM15 and SRSF1 expression, and this effect was further enhanced by combined ES‐Cu treatment. SRSF1 knockdown also reduced SRSF1 expression, whereas co‐transfection with both sh‐RBM15 and sh‐SRSF1 further suppressed SRSF1 expression (Figure 6D). As shown in Figure 6E,F, sh‐RBM15, sh‐SRSF1, and ES‐Cu treatment promoted apoptosis and inhibited the level of Ki‐67 compared to the control group. In addition, ES‐Cu treatment enhanced the effect of sh‐RBM15. More importantly, co‐transfection with sh‐RBM15 and sh‐SRSF1 further enhanced the number of TUNEL and reduced Ki‐67 positive cells. The expression of DLD and ATP7B decreased following SRSF1 and RBM15 knockdown. This inhibition had a synergistic effect with the ES‐Cu treatment. Furthermore, co‐transfection with sh‐RBM15 and sh‐SRSF1 further suppressed DLD and ATP7B expression (Figure 6G). Collectively, these results confirmed that RBM15 promoted tumor growth by mediating SRSF1.

## 4 Discussion

Copper ions are important trace elements essential for human activities and a potential target for anticancer therapy. In NSCLC, cuproptosis has been shown to play a role in tumorigenesis, progression, and treatment [17]. However, the regulatory mechanisms underlying cuproptosis in NSCLC have not been thoroughly investigated. The data in this study suggested that RBM15 could stabilize SRSF1 mRNA via the m6A reader YTHDF3 and that increased SRSF1 enhanced the ATP7B alternative splicing to inhibit cuproptosis in NSCLC. This presents new concepts for the diagnosis and treatment of NSCLC.

Cuproposis is a recently discovered mechanism of cell death that is closely related to oxidative phosphorylation and the lipoic acid pathway and shows great potential for anticancer therapy [18]. For example, cuproposis inhibits breast cancer cell growth [19]. In colorectal cancer, 4‐octyl itaconate has shown promising antitumor effects by promoting cupropsis [20]. In addition, copper‐related cancer treatment strategies can be categorized into two main categories [21]. Copper chelators are used to reduce the bioavailability of copper, thereby inhibiting the growth, recurrence, and metastasis of tumor cells. The second mechanism is to provide excess copper to induce cuproposis in cancer cells. Common copper chelators, such as TTM, selectively reduce copper ions in primary tumors and inhibit copper‐mediated metastasis while protecting adjacent healthy tissues [22]. Similarly, copper‐inducing agents (e.g., ES‐Cu) have shown promising therapeutic effects in various tumors and can inhibit the growth of tumor cells by inducing cuproposis [23]. In the present study, our results showed that ES‐Cu treatment inhibited cell proliferation and tumor growth, suggesting its potential as a therapeutic agent.

RBM15, a key regulator of m6A modification, promotes the migration, proliferation, and invasiveness of NSCLC cells [24]. For example, experiments investigating the effect of RBM15 on radioresistance in NSCLC cells showed that RBM15 overexpression significantly abrogated the inhibitory effects of radiation on the viability, proliferation, and invasive capacity of H520 cells and attenuated radiation‐induced apoptosis [25]. RBM15 overexpression also promotes the invasive, migratory, and proliferative abilities of A549 and H1734 cells [26]. Similarly, our study found that RBM15 knockdown effectively inhibited NSCLC cell migration, invasion, and proliferation, but RBM15 overexpression had opposite results. Multiple studies have shown a strong association between cuproptosis and m6A modification. YTHDF3 interacts with solute carrier family 31 member 1 (SLC31A1) to co‐regulate citrate synthesis in breast cancer [27]. In our study, we first reported that RBM15 knockdown enhanced the copper ion levels and inhibited the expression of the copper homeostasis‐related genes, DLD and ATP7B, which in turn induced copper homeostasis. These findings revealed a novel mechanism of action of RBM15 in NSCLC.

The effect of m6A modification on RNAs is mediated by m6A binding proteins. RBM15‐mediated m6A modification of BAX inhibitor motif containing 6 (TMBIM6) by recruiting IGF2BP3 to promote the progression of laryngeal squamous cell carcinoma [28]. METTL16 regulates alias B‐Myb (MYBL2B) mRNA m6A modification to promote cell cycle progression in hematopoietic stem and progenitor cells by mediating IGF2BP1 [29]. In this study, we showed that RBM15 facilitated the modification of SRSF1 through YTHDF3, and further studies revealed that RBM15‐mediated m6A modification of SRSF1 not only increased cell invasion and proliferation but also inhibited cuproptosis. Additionally, in vivo studies further validated that RBM15 facilitated tumor growth by regulating SRSF1. Furthermore, numerous studies have shown that about 35%–50% of the m6A peaks are concentrated in the 3′UTR [30]. Our predictions are consistent with this conclusion; the m6A modification sites in SRSF1 mRNA are mainly concentrated in the 3′‐UTR. However, there are differences between the predicted sites and the actual sequencing sites, which may be due to limitations in the prediction algorithm, resulting in different results for different algorithms, as well as antibody specificity issues in MeRIP Seq or false positives/negatives during peak call periods [31, 32]. In addition, cell type‐specific regulation or dynamic m6A modifications under specific conditions (e.g., stress) not covered by the training data [33]. These inconsistent sites may harbor novel regulatory mechanisms worthy of further investigation.

Additionally, SRSF1 is involved in regulating mRNA splicing and is an important splicing factor that contributes to gefitinib resistance and malignant progression in NSCLC [34]. SRSF1 also affects the chemosensitivity of NSCLC by regulating the selective splicing of caspase 9 [35]. Although our observations suggest that RBM15 inhibits cuproptosis by increasing the stability of SRSF1 mRNA, the mechanism underlying this process remains unclear. ATP7B is a copper transporter protein that is essential for maintaining copper homeostasis in vivo and is a critical factor that contributes to cuproptosis. ATP7B is associated with a reduced risk of lung cancer [36] and can serve as an indicator for predicting how patients will respond to platinum‐based chemotherapy [15, 37]. Consistent with these reports, we found that ATP7B‐S overexpression suppressed NSCLC cell viability and proliferation, promoted apoptosis, and inhibited DLD expression. Our results indicated that SRSF1 mediated the alternative splicing of ATP7B exon 21, affecting its transcript expression level, and that SRSF1 overexpression reversed the inhibitory effect of ATP7B‐S on the proliferation and invasion in A549 cells, which in turn inhibited cuproptosis.

In conclusion, our data provide a new perspective for inhibiting cuproptosis. RBM15 is anticipated to become a novel target for NSCLC treatment, thereby improving patient survival. However, we did not perform relevant experiments at the clinical level; the feasibility of this mechanism requires further exploration.

## Ethics Statement

All animal experiments were approved by the Animal Care and conducted according to the ethical standards of the animal experiment system approved by the Ethics Committee of Hainan Medical University (XSTS2025073).

## Conflicts of Interest

The authors declare no conflicts of interest.

## Supporting information


**Figure S1:** Knockdown of RBM15‐induced cell death was dependent on the cuproposis. sh‐RBM15 was transfected into A549 cells and treatment with TTM. (A) Cell viability was detected using CCK‐8 assay. (B) Transwell assay was utilized to evaluate the invasion ability. (C) Flow cytometry was used to evaluate cell apoptosis. (D) Western blotting was used to detect the DLD expression. Data are presented as mean ± SD (*n* = 3, **p < 0.01, ***p < 0.001).

**Figure S2:** YTHDF3 bound most strongly with SRSF1 mRNA. RIP experiments to verify the binding of YTHDF1/2/3, YTHDC1/2, and SRSF1 mRNA. Data are presented as mean ± SD (*n* = 3, **p < 0.01, ***p < 0.001).

**Figure S3:** RT‐qPCR was used to detect ATP7B‐L and ATP7B‐S expression in HBE1 and A549 cells (*n* = 3, *p < 0.05, **p < 0.01).

**Figure S4:** ATP7B‐s triggered cuproposis in A549 cells. Overexpressed ATP7B‐s was transfected into A549 cells and treatment with TTM. (A) Cell viability was detected using CCK‐8 assay. (B) Transwell assay was utilized to evaluate the invasion ability. (C) Flow cytometry was used to evaluate cell apoptosis. (D) Western blotting was used to detect the DLD expression. Data are presented as mean ± SD (*n* = 3, *p < 0.05, **p < 0.01, ***p < 0.001).

## Data Availability

Data sharing is not applicable to this article as no new data were created or analyzed in this study.
