# Supplementary figures and images for: RBM15 Mediated m6A Modification of SRSF1 Inhibits Cuproptosis in Non‐Small Cell Lung Cancer by Mediating ATP7B Alternative Splicing
Source: Kaohsiung J Med Sci. 2025 Sep 9;42(1):e70098. doi: 10.1002/kjm2.70098 (PMC12782256; doi:10.1002/kjm2.70098)

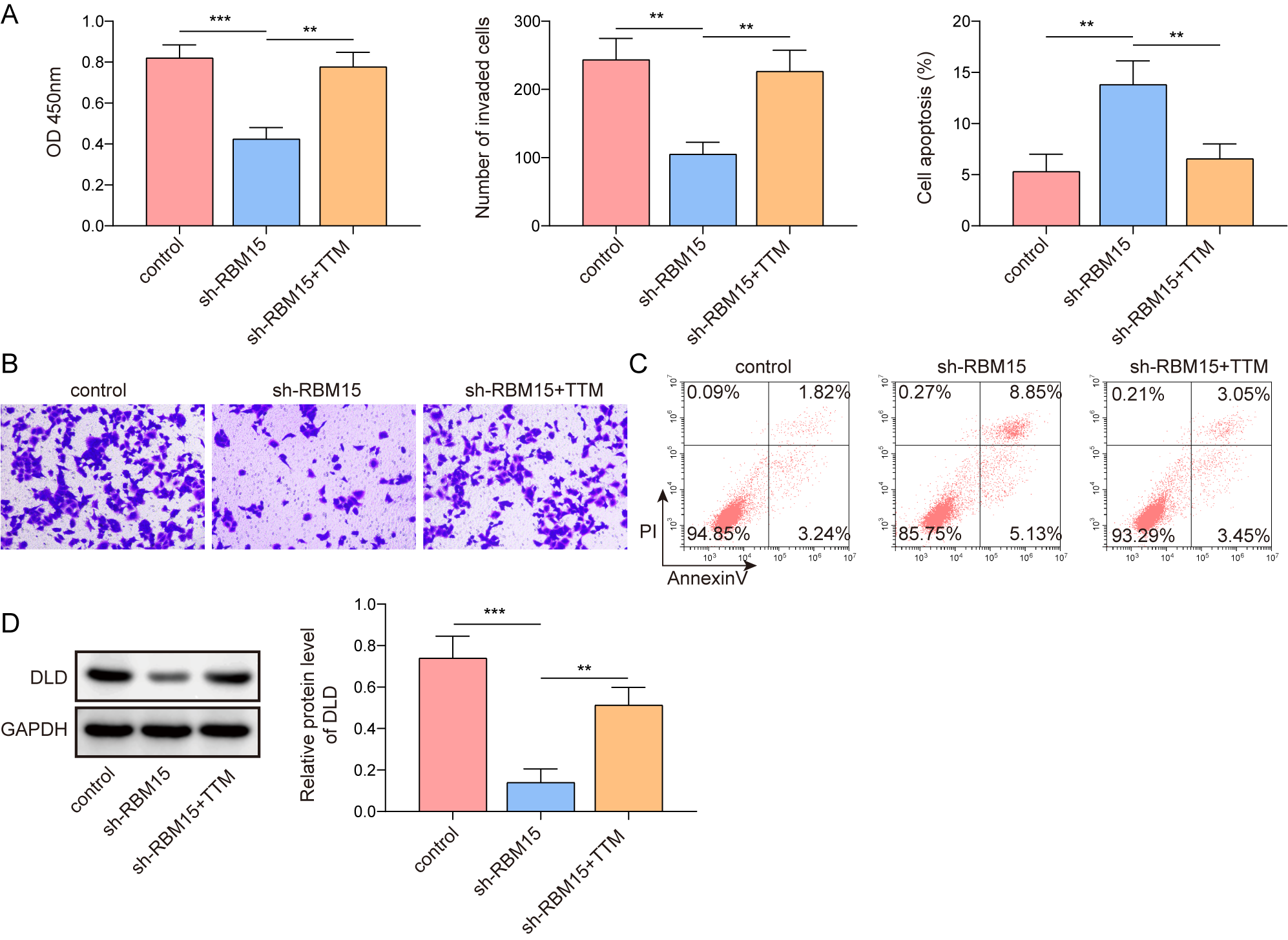

Supplement: Supplementary file 1 — Figure S1: Knockdown of RBM15‐induced cell death was dependent on the cuproposis. sh‐RBM15 was transfected into A549 cells and treatment with TTM. (A) Cell viability was detected using CCK‐8 assay. (B) Transwell assay was utilized to evaluate the invasion ability. (C) Flow cytometry was used to evaluate cell apoptosis. (D) Western blotting was used to detect the DLD expression. Data are presented as mean ± SD (n = 3, **p < 0.01, ***p < 0.001). [file KJM2-42-e70098-s002.tif]

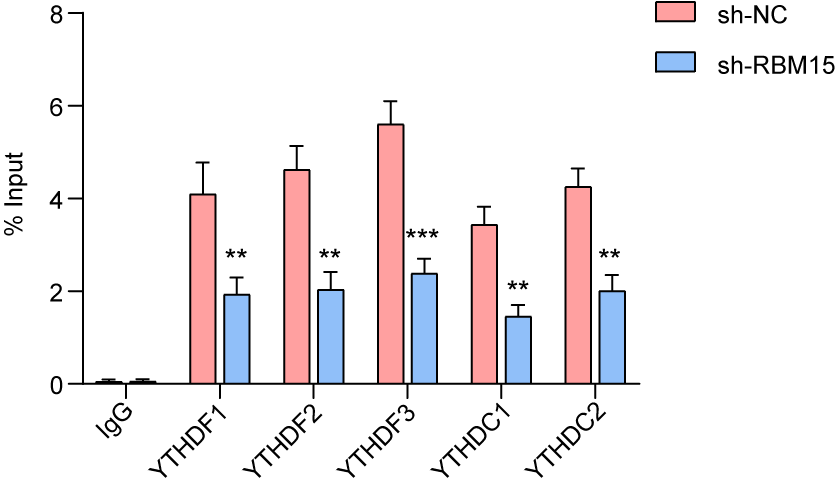

Supplement: Supplementary file 2 — Figure S2: YTHDF3 bound most strongly with SRSF1 mRNA. RIP experiments to verify the binding of YTHDF1/2/3, YTHDC1/2, and SRSF1 mRNA. Data are presented as mean ± SD (n = 3, **p < 0.01, ***p < 0.001). [file KJM2-42-e70098-s001.tif]

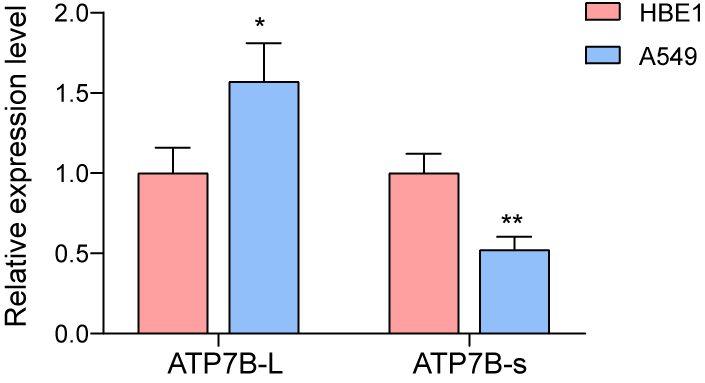

Supplement: Supplementary file 3 — Figure S3: RT‐qPCR was used to detect ATP7B‐L and ATP7B‐S expression in HBE1 and A549 cells (n = 3, *p < 0.05, **p < 0.01). [file KJM2-42-e70098-s003.tif]

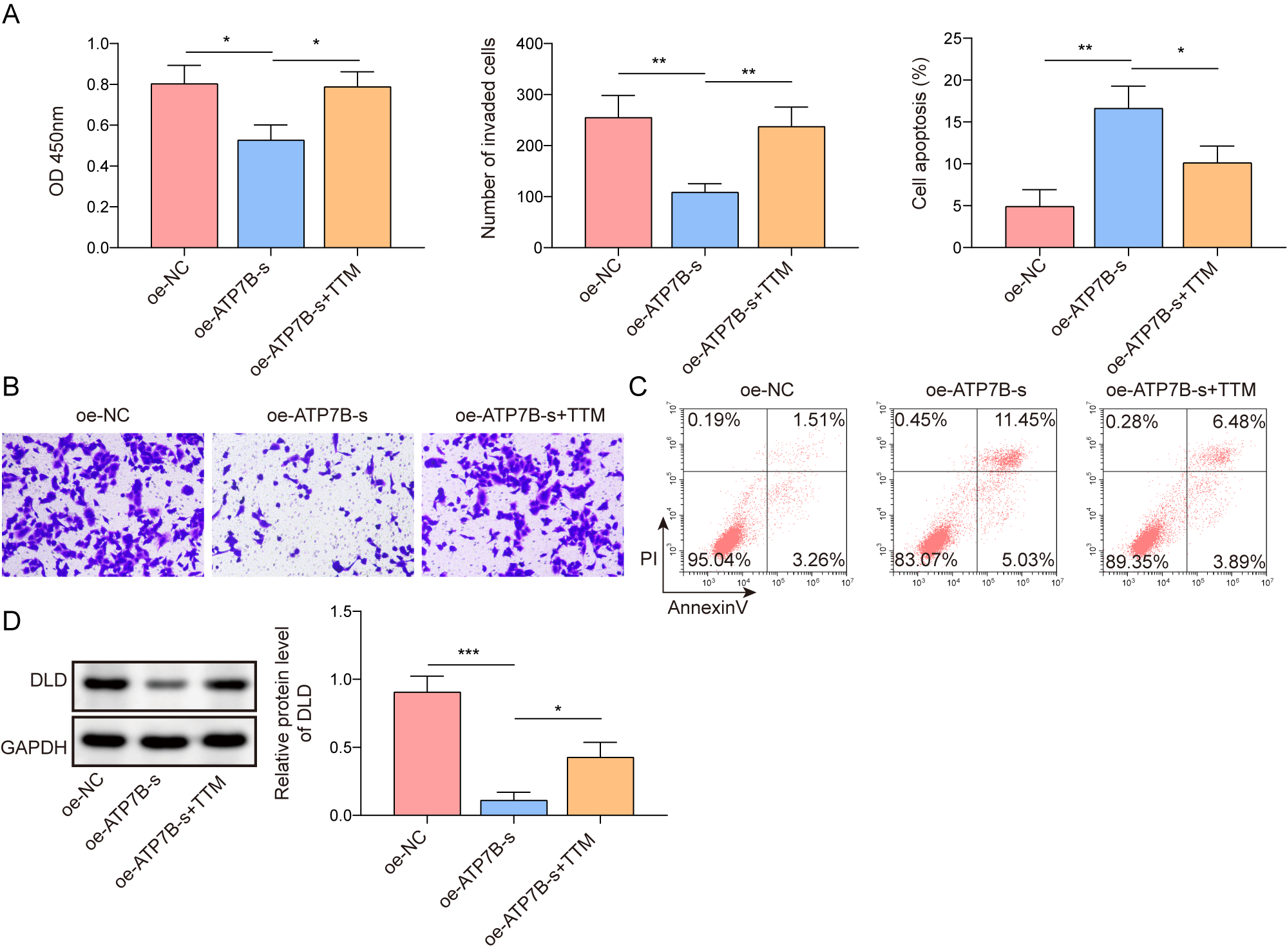

Supplement: Supplementary file 4 — Figure S4: ATP7B‐s triggered cuproposis in A549 cells. Overexpressed ATP7B‐s was transfected into A549 cells and treatment with TTM. (A) Cell viability was detected using CCK‐8 assay. (B) Transwell assay was utilized to evaluate the invasion ability. (C) Flow cytometry was used to evaluate cell apoptosis. (D) Western blotting was used to detect the DLD expression. Data are presented as mean ± SD (n = 3, *p < 0.05, **p < 0.01, ***p < 0.001). [file KJM2-42-e70098-s004.tif]
